# Study of the micro-climate and bacterial distribution in the deadspace of N95 filtering face respirators

**DOI:** 10.1038/s41598-018-35693-w

**Published:** 2018-11-26

**Authors:** Quan Yang, Hui Li, Shengnan Shen, Guoqing Zhang, Ruiyang Huang, Yong Feng, James Yang, Shiyue Ma

**Affiliations:** 1 The Institute of Technology Sciences, Wuhan University, Wuhan, China; 2 School of Power and Mechanical Engineering, Wuhan University, Wuhan, China; 3 College of Life Science, Wuhan University, Wuhan, China; 4 School of Basic Medical Sciences, Wuhan University, Wuhan, China; 5 Department of Mechanical Engineering, Texas Tech University, Lubbock, TX USA; 6 School of mechanical and energy engineering, Tongji University, Shanghai, China

## Abstract

It is common for people to use N95 filtering facepiece respirators (FFRs) in daily life, especially in locations where particulate matter (PM_2.5_) concentration is rising. Wearing N95 FFRs is helpful to reduce inhalation of PM_2.5_. Although N95 FFRs block at least 95% of particles from the atmosphere, the deadspace of N95 FFRs could be a warm, wet environment that may be a perfect breeding ground for bacterial growth. This work studies the micro-climate features including the temperature distribution and water vapor condensation in the deadspace of an N95 FFR using the computational fluid dynamics (CFD) method. Then, the temperature and relative humidity inside the same type of N95 FFR are experimentally measured. There is a good agreement between the simulation and experimental results. Moreover, an experiment is conducted to study the distribution of bacteria sampled from the inner surface of an N95 FFR after donning.

## Introduction

At a specific pressure, water vapor condensation may occur when the ambient temperature is lower than the condensing temperature. Many researches have studied this physics process. Morrison *et al*.^1^ focused on the heat transfer of single phase condensation. Groff *et al*.^2^ studied the condensation of vapor-gas mixture, and reported that the *k-ε* turbulence model for both the film and the mixture flows produced the best agreement. Fronk^3^ accurately measured heat transfer coefficients and pressure drop during the condensation of CO_2_ in a rectangular channel with hydraulic diameter ranging from 0.10 mm to 0.16 mm and an aspect ratio in the range of 1 to 4 at reduced pressures of 0.69, 0.78 and 0.87 (*T*_sat_ = 15 °C, 20 °C and 25 °C). Zschaeck *et al*.^4^, Ambrosini *et al*.^5^ and Dehbi *et al*.^6^ used different models to simulate water vapor condensation inside industry equipment and experimentally verify the results. Rosa *et al*.^7^ summarized the study of gas condensation in the industry in their review paper. The N95 FFR has been commonly used to protect people from air pollution in their daily life. However, water vapor from the human respiratory system can condense on the inner surface of the N95 FFR, especially in the winter time. This event combined with the occurrence of poor heat dissipation could lead to a feeling of stuffiness during extended periods of wear. Moreover, this wet and warm region inside the N95 FRRs will inevitably promote bacterial growth. As for the study of the N95 FFR’s filtering function, Macintyre^8^ experimentally found that N95 FFRs were protective against outside bacteria in the living environment. The Air Force Research Laboratory Airbase Technologies Division (AFRL/RXQ) examined the effectiveness of three energetic decontamination methods (ultraviolet germicidal irradiation, microwave-generated steam, and moist heat) on two National Institute for Occupational Safety and Health-certified N95 FFRs (3M models 1860s and 1870) contaminated with H5N1. These methods effectively decontaminated H5N1 on the two FFR models tested and did not drastically affect their filtering function, however, other considerations may influence decisions to reuse FFRs^9^. Previously, we have studied N95 wear pattern and quality of comfort and fit, such as contact characteristics between a respirator and a headform, customized design and 3D printing of a face seal, as well as a ventilation fan design to dissipate the heat and CO_2_ for increasing the comfort of wearers^10–13^.

This work studies micro-climate features in the deadspace of N95 FFRs. It investigates the distribution of temperature, volume fraction of water vapor, and liquid water inside the FFR using the computing fluid dynamic (CFD) method with a self-developed user defined function (UDF) as well as experimental measurement. Results of the simulation calculation are in accordance with test data. In addition, an experiment is conducted to study the distribution of bacteria sampled from the inner surface of an N95 FFR after donning.

## CFD modeling of water vapor condensation

### Simulation model

**Figure 1:**
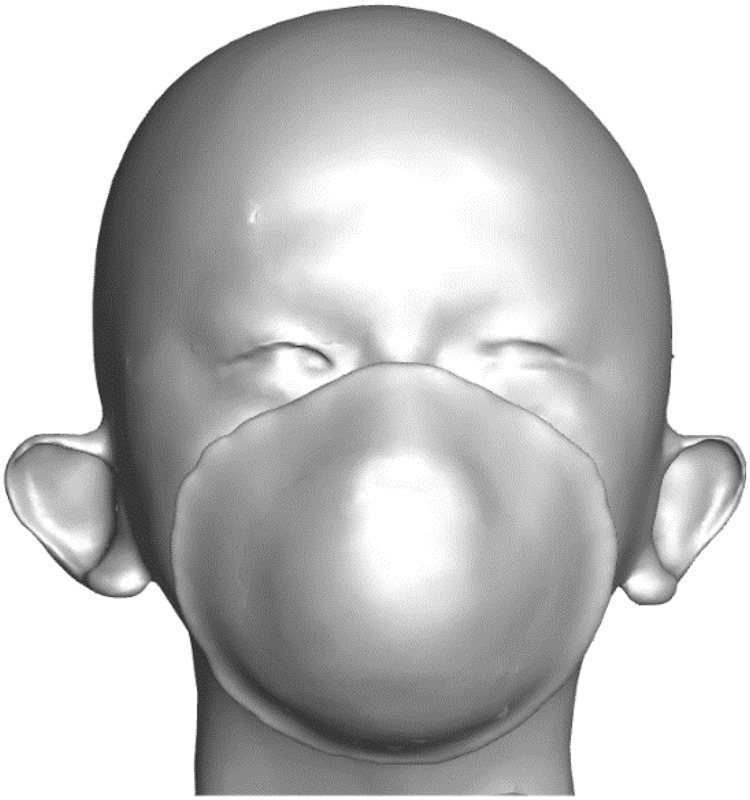
The three-dimension geometric model of a head wearing an N95 FFR used in this work.

**Figure 2:**
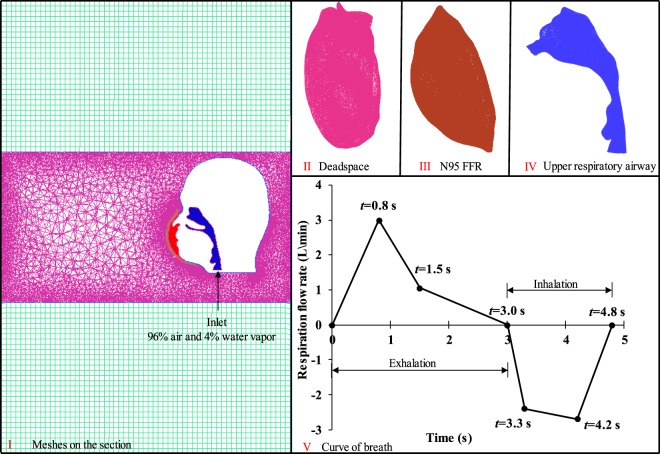
Four parts of the mesh and respiration flow rate^14^.

A three-dimension geometric model of a head wearing an N95 FFR is shown in Fig. 1, which is based on our previous study^11^. Computing meshes of the fluid domain generated by ANSYS ICEM CFD 14.0 is shown in Fig. 2, and the reliability of the mesh was verified previously^14^. In this paper, the N95 FFR dense meshes were used to catch more flow detail. The deadspace (II), N95 FFR (III) and the upper respiratory airway (IV) were discretized into tetrahedral elements, which amount to 142,831, 127,766 and 121,654, respectively. Meanwhile, unstructured tetrahedral meshes are generated for the upper surrounding region around the head, and structured meshes were generated for the lower surrounding region. The velocity inlet was set at the bottom of the upper respiratory airway. In the simulation, the saturation temperature of water was assumed to maintain 373 K because the variation of pressure was not large enough to affect the saturation temperature. Simultaneously, the latent heat of vaporization was presumed to be 2257.6 kJ/kg^15^. Furthermore, re-condensation and re-evaporation occur under appropriate conditions.

This study was approved by the Human Subjects Review Board of Wuhan University, the subject provided informed oral and written consent, and all methods were performed with the relevant guidelines and regulations.

### CFD-setup and boundary conditions

The transient analysis simulation was performed using ANSYS FLUENT 17.0. The standard *k-ε* and multiphase were applied to the entire meshing, and energy change was also taken into account. Jackie^16^ revealed that steady inhalation would not increase the complexity and post-processing of the simulation, and the respiration flow rate was given. The velocity at the inlet was simplified into successive segments, as shown in Fig. 2. The breath cycle included both exhalation and inhalation, and we assumed that exhalation occurs first. The velocity direction was assumed to be perpendicular to the inlet throughout the entire simulation. The gases of the inlet were considered to be a mixture of air and water vapor, with the volume ratio set at 96% and 4%, respectively. In addition, the ambient temperature of the simulation was 296.25 K, while the temperature of the inlet airflow was set at 307.6 K^17^. Two different materials were assigned to the human face and the surface of the N95 FFR, as shown in Table 1. For the fluid domain, material properties of air, water vapor, and water were set in accordance with the ANSYS FLUENT material library. The N95 FFR was set to be a porous zone with a fluid porosity of 0.88, and its viscous resistance in all three directions was 1.12 × 10^10^ ^18,19^.

**Table 1:** Material properties of N95 FFR and face^20,21^.

Material	Density (kg/m^3^)	Cp (Specific Heat) (J/(kg·K))	Thermal Conductivity (W/(m·K))
N95 FFR	910	1715	0.05
Face	1026	3135	0.39

**Figure 3:**
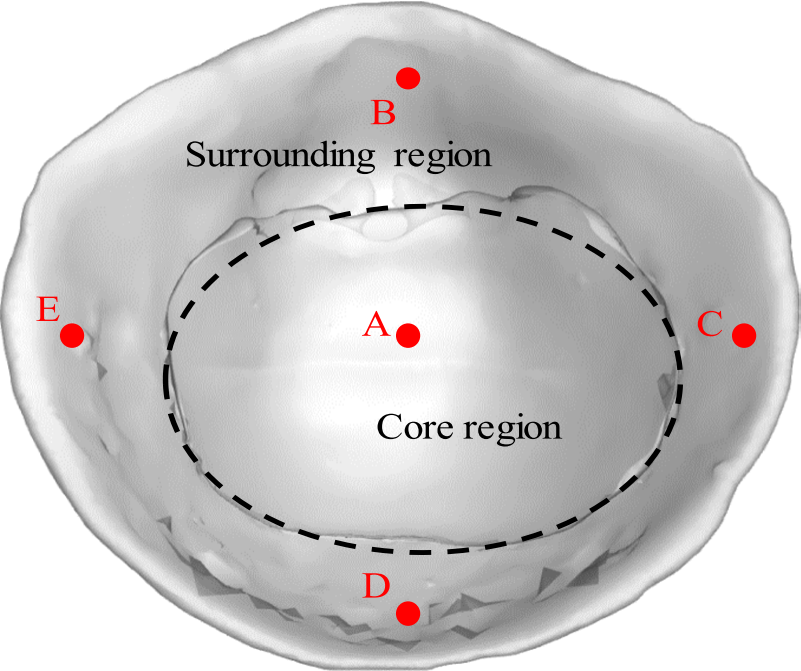
The positions of five monitoring points and two divided regions of the N95 FFR.

The time duration of a single breath cycle was 4.8 s, with a simulation time step of 0.05 s and a total simulation time of 1,600 s. We set five monitoring points above the inner surface of FFR to record the temperature history with respect to time. The position of five monitoring points is shown in Fig. 3. For convenience, the central part of the deadspace is called the core region of FFR deadspace, while the remaining portion surrounding the core region is called the surrounding region of FFR deadspace.

### Simulation results

**Figure 4:**
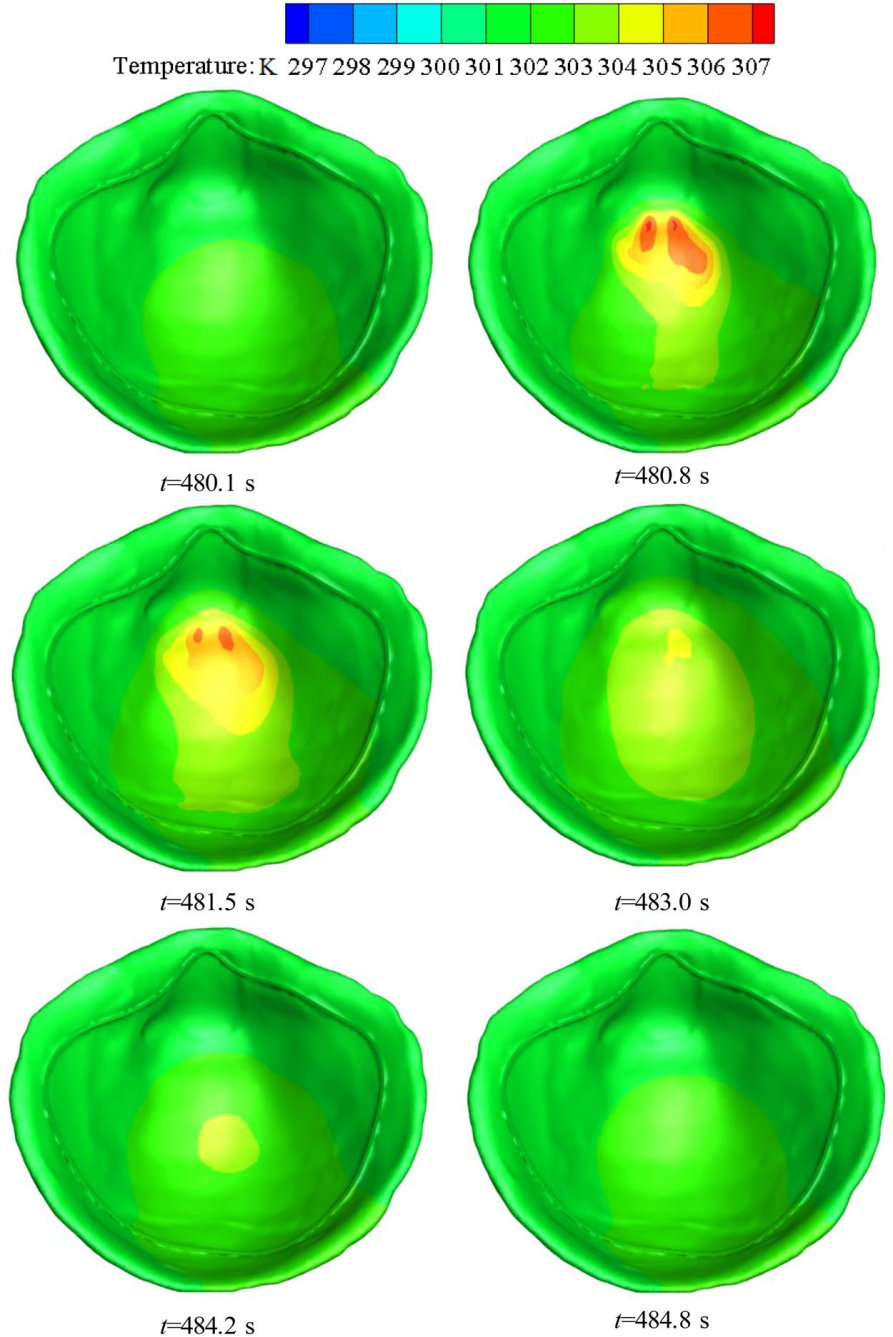
Temperature contours of N95 FFR in the 101st breath cycle.

**Figure 5:**
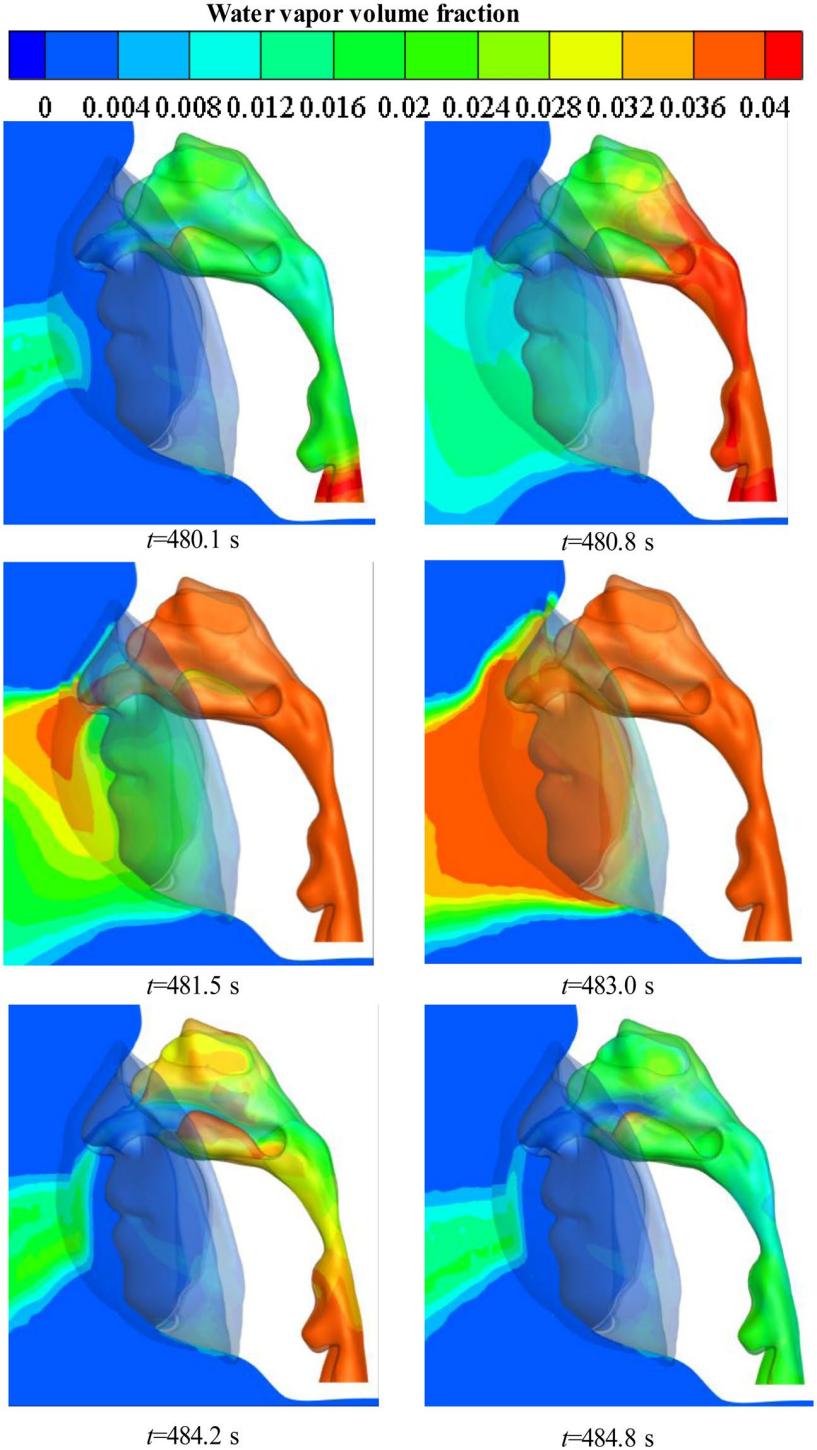
Water vapor volume fraction contours on the cross-section in the 101st breath cycle.

The CFD simulation in the deadspace of the N95 FFR was performed. Figure 4 shows the temperature distribution on the inner surface of the FFR during the 101st breath cycle at six key time points shown in Fig. 2. It can be found that there is an even temperature distribution on the inner surface of the N95 FFR as the new breath cycle starts, and the temperature of the core region then increases significantly during exhalation, reaching a peak of 307 K, which is nearly equal to the temperature of the inlet flow. During inhalation, the temperature of the core region decreases gradually until the end of the breath cycle. Meanwhile, the temperature of the core region is slightly higher than that of the surrounding region because the core region is closer to the nostrils. Overall, the temperature of deadspace is approximately 6 K higher than the ambient temperature of 296.25 K. Figure 5 shows the cross-sectional view of the water vapor volume fraction in the upper respiratory system during the 101st breath cycle. It can be found that the water vapor volume fraction around the FFR rises rapidly during exhalation, and the largest concentration of water vapor occurs at the core region at 483.0 s (end of exhalation). It is apparent that the concentration of water vapor decreases during inhalation.

**Figure 6:**
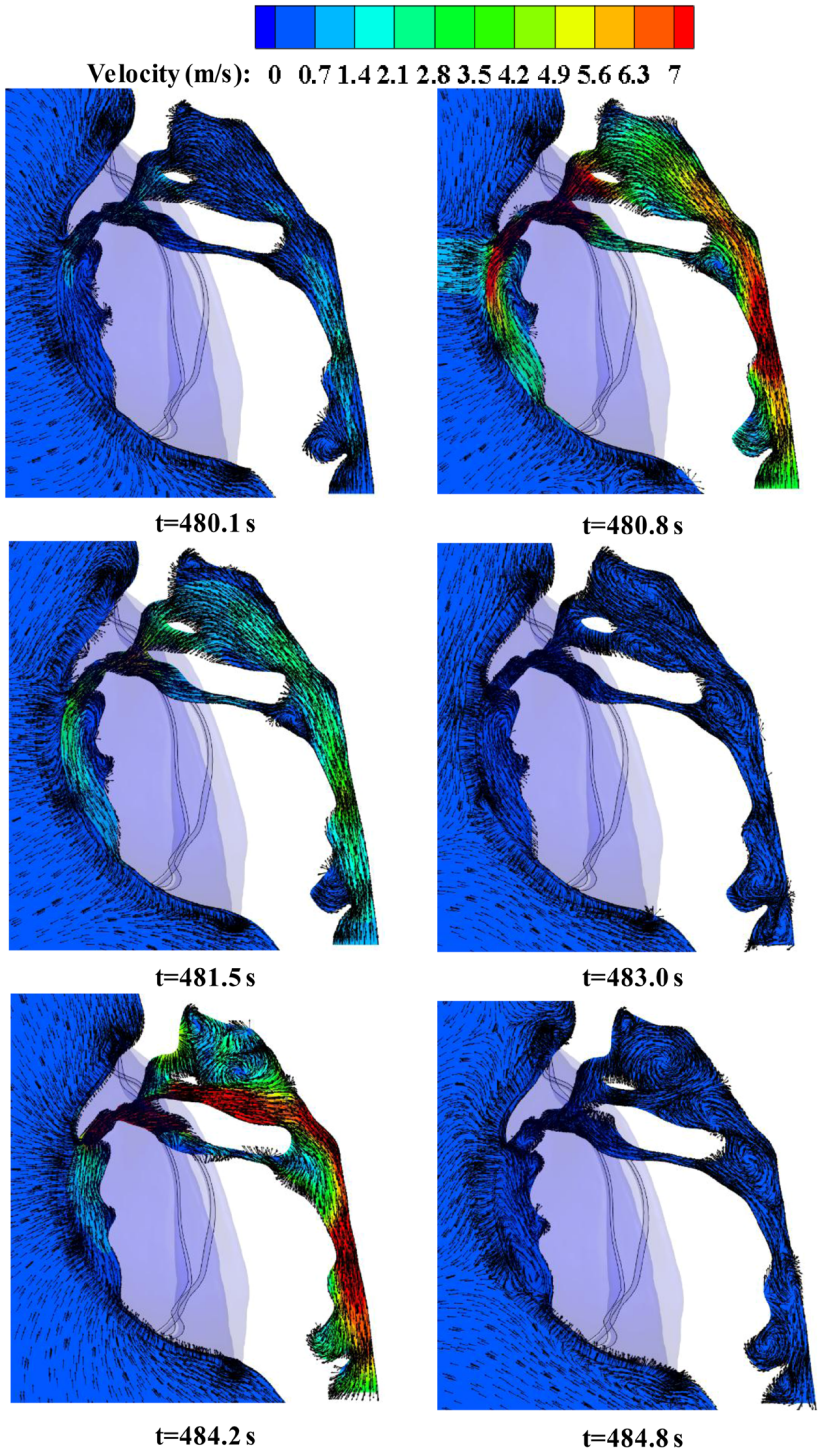
Velocity vector contours of airflow on the cross-section in the 101st breath cycle.

Figure 6 shows the cross-sectional view of airflow velocity in the upper respiratory system, with the arrows indicate the orientation of breath gases. It is found that exhaled gases flow down along the FFR inner surface during exhalation. Meanwhile, the directional arrows for gas flow show that there is more water vapor in the center and bottom of the N95 FFR deadspace as shown in Fig. 5. On the other hand, when fresh air is inhaled from the nose to the upper respiratory tract, obvious eddy flows are observed because of the rapid increase of the tract size.

**Figure 7:**
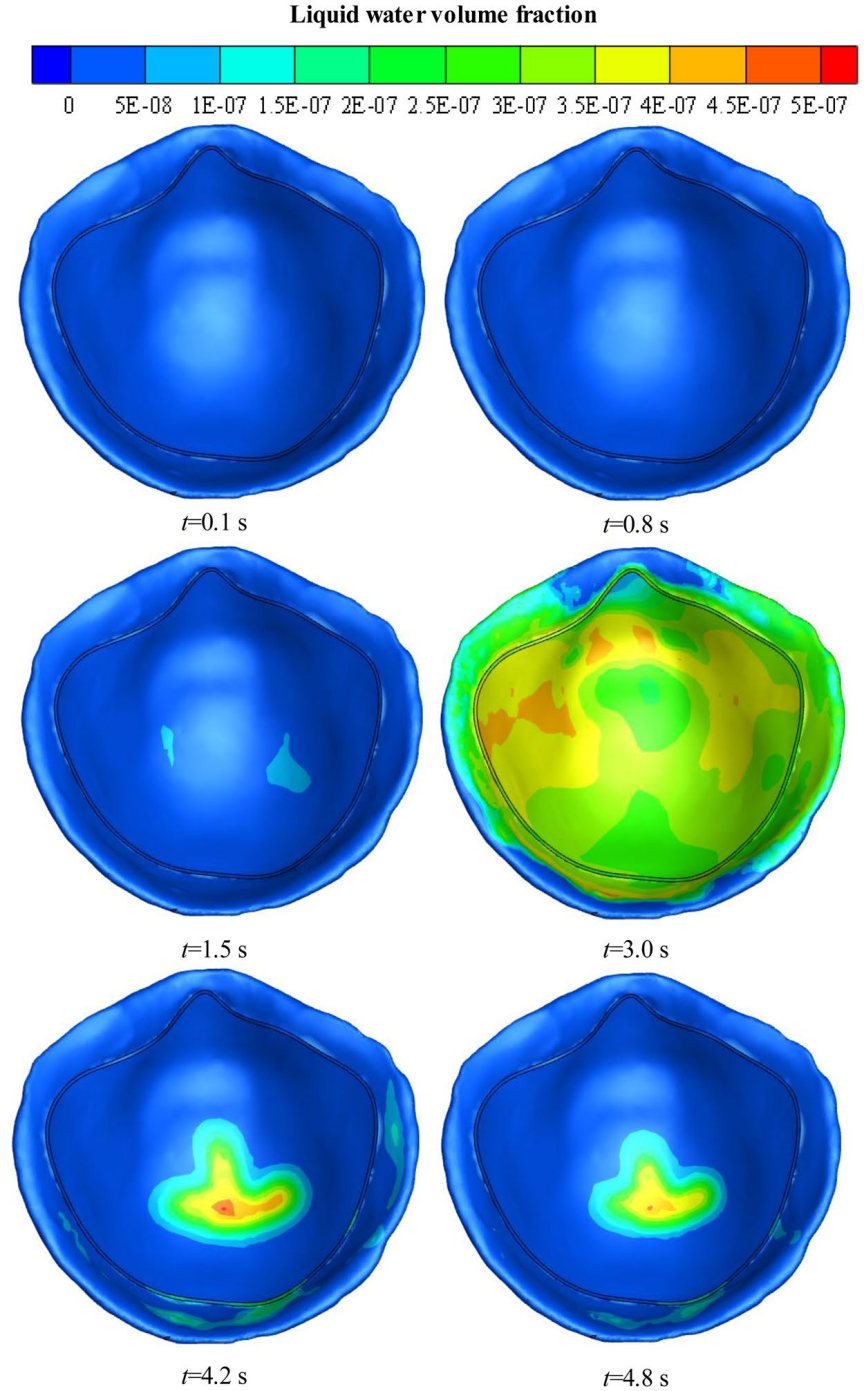
Liquid water volume fraction contours on the inner surface of N95 FFR in the first breath cycle.

Figure 7 shows the distribution of liquid water on the inner surface of the FFR within the first breath cycle at various time points. During exhalation, there is almost no liquid water at first because of limited water vapor, but then the liquid water begins to accumulate on the inner surface of the N95 FFR, reaching a peak of 4 × 10^−7^ at 3 s. The liquid water is distributed thoroughly on the inner surface of the N95 FFR. During inhalation, the liquid water on the inner surface of the N95 FFR decreases dramatically because there is no more water vapor being supplied and the re-evaporation of the liquid water occurs. At the end of the breath cycle, there is some remaining liquid water at the core region.

**Figure 8:**
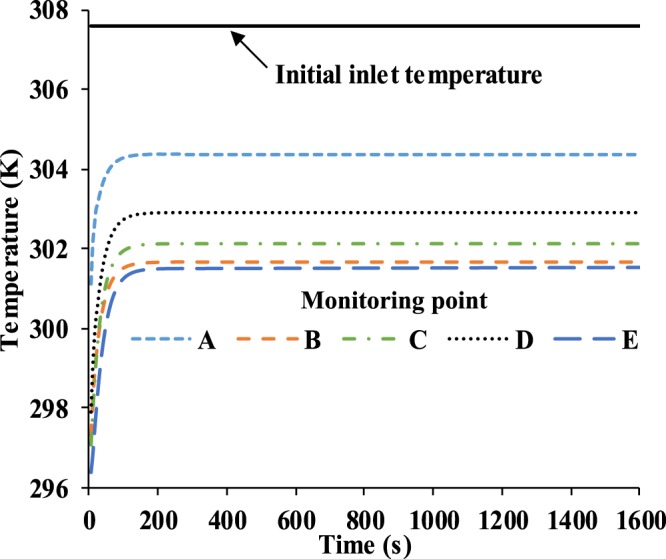
Temperature of five monitoring points in 1,600 s.

The temperature profiles of five monitoring points in the FFR deadspace during the entire breathing process (1,600 s) are shown in Fig. 8. The simulation time interval is 4.8 s, which is equal to the duration time for a single breathing cycle, and the data values are the average temperature in each breath cycle. Because of the relatively far distance from the nose, the temperature at monitoring points B, C, D and E, which rise from 296.25 K to approximately 301.6 K, 302.1 K, 302.9 K and 301.5 K, respectively, are slightly lower than the temperature of monitoring point A. Meanwhile, monitoring point A located at the center of the core region of FFR deadspace has a rapid increase of temperature to approximately 304.3 K, but it is still 3.3 K lower than the initial inlet temperature of 307.6 K. This means there is a heat dissipation from both the surrounding and core regions of the FFR deadspace to the outside of the FFR during a human breath. The resultant temperature reduction is approximately 6 K at four monitoring points on the surrounding region and 3.2 K at monitoring point A, which is at the core region.

**Figure 9:**
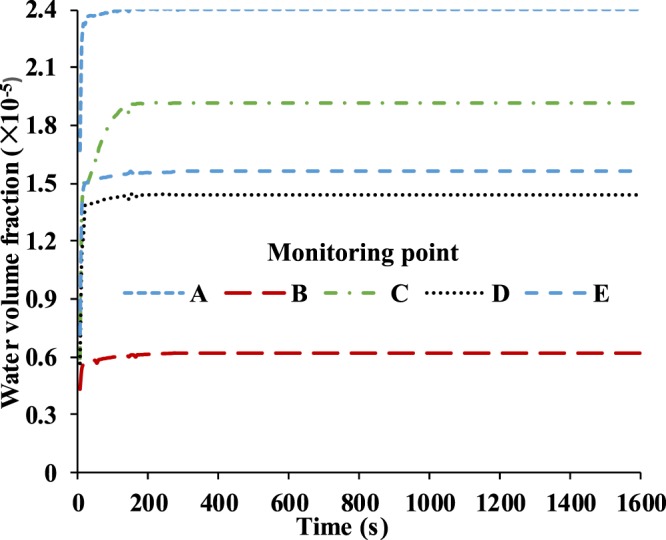
Water volume fraction of five monitoring points in 1,600 s.

Water volume fraction profiles are presented similarly to temperature results. As Fig. 9 shows, the water volume fraction of monitoring point A has the largest value among the five monitoring points due to its specific position, which is the closest to the nostrils, so there is the most water vapor being supplied at monitoring point A. In contrast, the water volume fraction of monitoring point B has the smallest value among five monitoring points because it is above the nose which is opposite to the direction of the flow from the nostrils. On the whole, the values of liquid water volume fraction range from 0.6 × 10^−5^ to 2.4 × 10^−5^, and we can conclude that there will be more liquid water condensing on the N95 FFR inner surface because it is subjected to direct exhaled airflow.

## Experiment and Comparison

**Figure 10:**
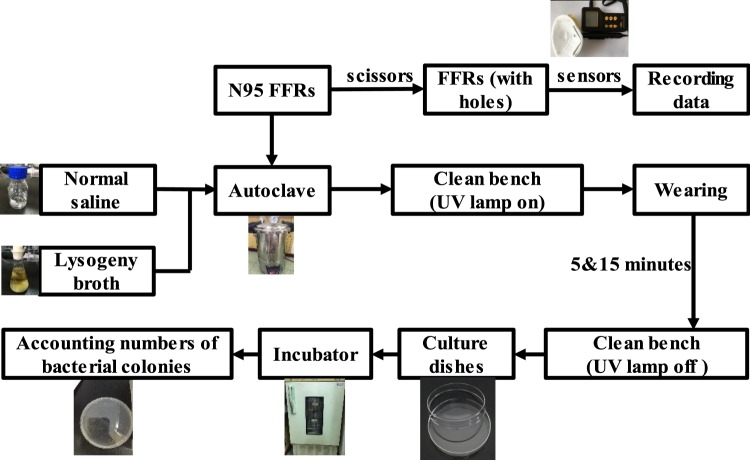
Flow diagram of experiment.

The above simulation work presents the distribution of temperature and water vapor condensation on the inner surface of the FFR during a human breath. Next, the experimental measurement of the micro-climate inside the FFR is performed. The entire experimental flow diagram is depicted in Fig. 10. It includes two parts: (1) the measurement of temperature and relative humidity inside the FFR and (2) the measurement of bacteria distribution on the inner surface of the FFR.

### Experimental measurement of temperature and relative humidity inside the FFR

**Figure 11:**
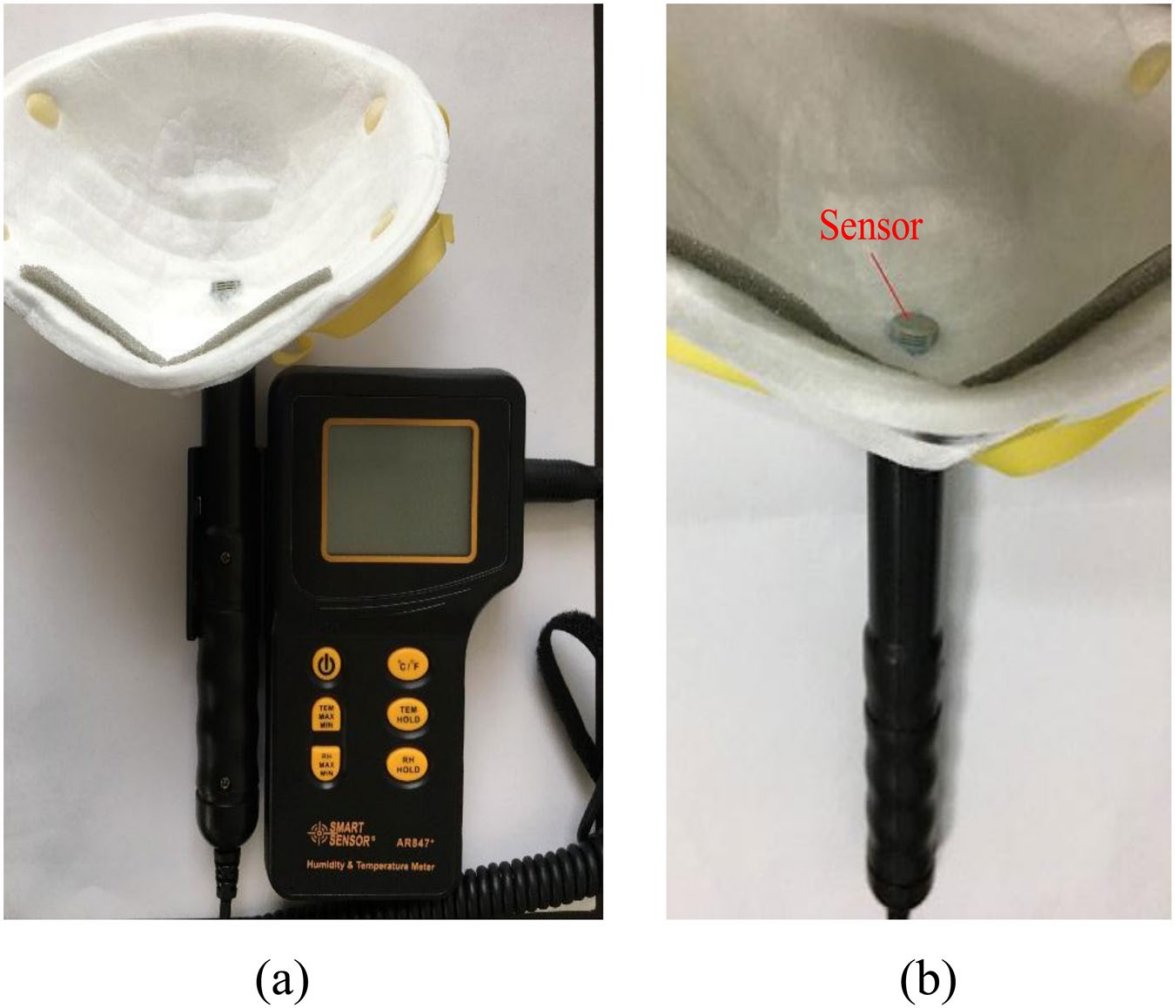
The FFR and instrument used in the experiment: (**a**) FFR contacting with the apparatus, (**b**) temperature/humidity sensor attached inside FFR.

The approach used to measure the temperature and relative humidity inside the FFR is shown in Fig. 11. Scissors were used to cut a hole in an N95 FFR (3M^TM^ 8210CN, 3M Corp., St. Paul, MN, USA), and the size of the hole approximately matched the dimensions of the detector of the SMART SENSOR AR847+ (Arco Science & Technology LTD., Dongguan, Guangdong Province, China), which was used to record the temperature and relative humidity with respect to time (resolution: 0.1 K and 0.1% RH). The detector was kept tangent to the local inner surface of the N95 FFR to avoid contact with face skin. Airflow leakage from the hole was avoided by sealing the gap between the detector and the N95 FFR. A previous study has shown that the resistance of respiration only increases significantly under proper humidity after 4 hours^15,16^. Therefore, we performed a 15-minute experimental measurement by assuming that the resistance of respiration remains unchanged. We repeated each measurement five times for the five monitoring points as shown in Fig. 3. The air conditioning was turned on to keep the indoor environment steady. The ambient temperature and relative humidity were maintained at 296.25 K and 34.4% RH, respectively.

**Figure 12:**
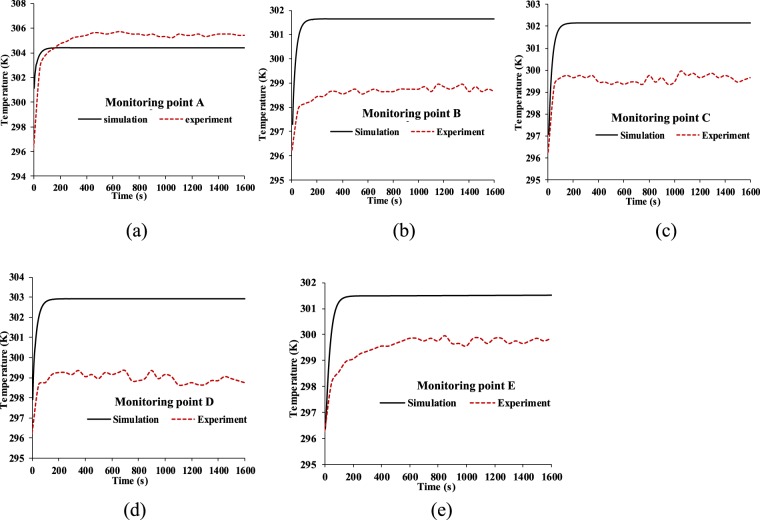
Temperature comparison between simulation and the experiment on monitoring point: (**a**) A, (**b**) B, (**c**) C, (**d**) D, (**e**) E.

Figure 12 shows the temperature comparison between the simulation and experiment, where the experimental data were taken at five monitoring points every 50 seconds. It is found that the experimental temperature of monitoring point A is about 1 K higher than the temperature of the simulation. Meanwhile it is found that the experimental temperature of the four monitoring points are 2 K to 3 K lower than those of the experiment. The reason is that the N95 FFR is set to be an ideal porous medium in CFD, which could lead to an ideal heat dissipation of the N95 FFR, so the heat of the N95 FFR core region can easily be transferred to the surrounding region as Fig. 4 depicts.

**Figure 13:**
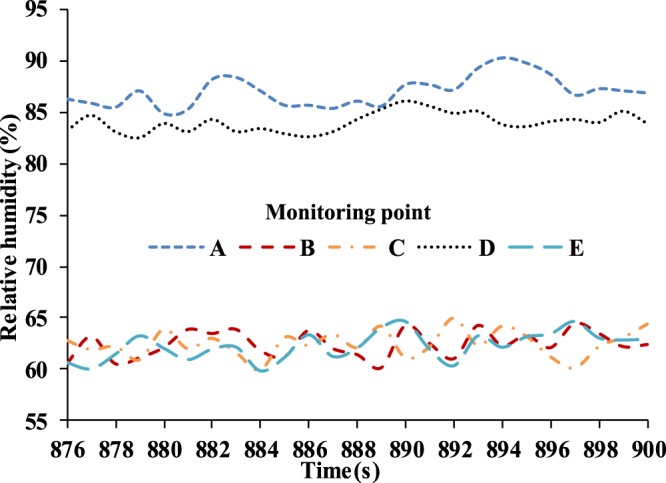
Experimental results of relative humidity inside FFR (sampling interval: 3 s).

Figure 13 shows experimental results of the relative humidity above five monitoring points from 876 s to 900 s since the complete experimental data is too large. Here, the sampling time interval is 3 s. It is found that the values for the relative humidity fluctuate with exhalation and inhalation. Due to the position and direction of the nostrils, the relative humidity at monitoring points A and D are slightly higher than those at monitoring points B, C, and E. Moreover, the measured relative humidity inside the FFR is approximately 1.5~2.6 times higher than the initial ambient relative humidity of 34.4%. This will make people feel uncomfortable to some extent. In addition, this warm and wet micro-climate could contribute to the growth and activity of bacteria.

### Experimental measurement of bacteria distribution on the inner surface of the FFR

To identify the bacteria distribution inside the FFR, we used three identical N95 FFRs that are the same as the ones used in the previous experiment. As Fig. 10 shows, we first prepared 100 ml normal saline and 500 ml lysogeny broth, and then we disinfected the normal saline, lysogeny broth, and three N95 FFRs in the autoclave. Next, each of the N95 FFRs was worn for 5 minutes and 15 minutes. Finally, we extracted the bacteria from the N95 FFRs and determined the bacteria quantity.

First, three N95 FFRs were sealed in a container to avoid outside water vapor entering. Then the container, normal saline, and lysogeny broth were placed into the autoclave (SHENAN DSX-280A (Shanghai Medical Instrument Co., Ltd., Shanghai, China)) for one hour. The working temperature and pressure of the autoclave were set at 105 kPa and 394.15 K, respectively. Then, they were immediately transferred from the autoclave to the clean bench. The ultraviolet lamps in the clean bench remained turned on for 30 minutes to kill any possible bacteria coming from the ambient environment during transfer.

Secondly, the bacteria on three worn N95 FFRs were extracted and cultured. We prepared the lysogeny broth in the culture dishes, which solidified in a few minutes. Next, 500 μl normal saline was transferred from the conical flask to the centrifuge tube using a pipette, and another eleven identical centrifuge tubes were prepared in the same way. The previously mentioned preparation was done on the clean bench. Then, a volunteer (male, 24 years-old, 170 cm/55 kg) wore two prepared N95 FFRs for 5 and 15 minutes. The third N95 FFR was put on the clean bench as a reference.

Thirdly, we cut out five samples from two worn FFRs corresponding to the five monitoring points in Fig. 3, and only one sample from the reference FFR (the third one). A total of eleven samples were placed into the eleven centrifuge tubes filled with 500 μl normal saline, respectively. The twelfth centrifuge tube contains only 500 μl normal saline served as the reference. All twelve centrifuge tubes were shaken sufficiently to disperse the bacteria in the normal saline completely, and we obtained the bacterial liquid. Next, we transferred 100 μl of bacterial liquid in each centrifuge tube to three culture dishes. Finally, a total of 36 culture dishes were placed into the incubator, and the working temperature was set at 310.25 K. After a 24-hour cultivation, the bacterial colonies on each culture dish were counted.

**Figure 14:**
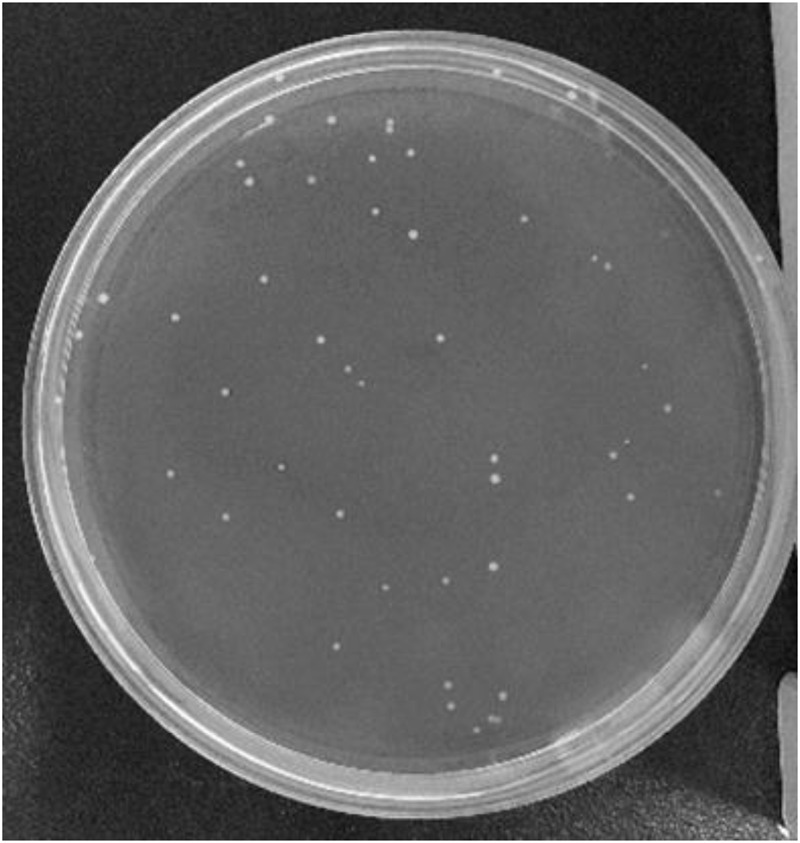
Bacterial colonies on the culture dish.

**Figure 15:**
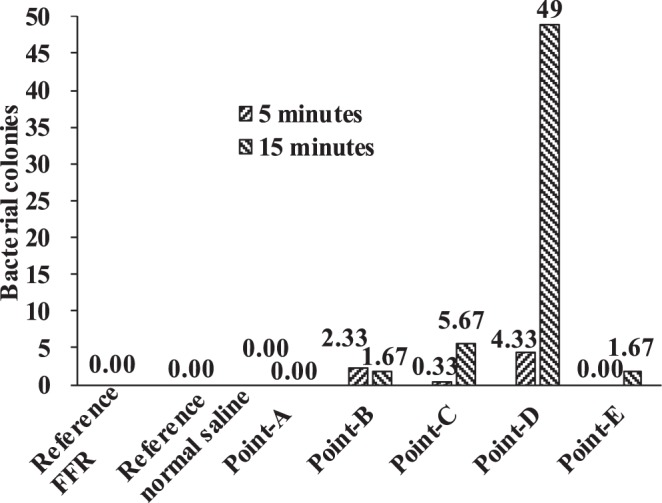
Numbers of bacterial colonies on the inner surface of FFR.

Finally, the bacterial colonies were counted as shown in Fig. 14 (one of 36 culture dishes), where a white dot represents a bacterial colony. The statistical information is shown in Fig. 15, and each number of bacterial colonies is the average of three culture dishes.

It was found that the number of bacterial colonies of the reference normal saline and reference FFR was zero (Fig. 15), indicating that the experimental process was reliable. Moreover, there are no bacterial colonies at the core region of the FFR but colonies exist at the surrounding region of the FFR. For example, no bacterial colony was observed at monitoring point A after 5 minutes or 15 minutes. It indicates that the bacterial colonies in this experiment originated from face skin, but not from human respiration system for healthy individuals. Additionally, the observed bacterial colonies did not move during respiration as the number of bacterial colonies at monitoring point A remained zero after 15 minutes.

Furthermore, the numbers of bacterial colonies were different at monitoring point C and E due to asymmetric wear. The largest number of bacterial colonies was observed at monitoring point D after 15-minutes of wear, since there is a larger relative humidity compared to other monitoring points as shown in Fig. 13. Actually, both monitoring points A and D have a large relative humidity (Fig. 13), which could encourage the growth of bacterial colonies. However, there were no original bacterial colonies at monitoring point A because there was no contact with face skin, which this study has shown may be responsible for bacterial growth.

## Conclusion

In this work, the micro-climate features of water vapor condensation and temperature distribution in the deadspace of the N95 FFR during exhalation and inhalation were studied by use of CFD simulation and experimental investigation. An experiment was conducted to study the distribution of bacteria sampled from the inner surface of N95 FFR after wearing for 5 and 15 minutes.

Simulation results show that the water vapor volume fraction around the FFR rises rapidly during exhalation, and that the largest concentration of water vapor is located in the core region at the end of exhalation. During inhalation, the concentration of water vapor significantly decreases. In addition, during inhalation, the liquid water on the inner surface of N95 FFR dramatically decreases because there is no additional water vapor being supplied and the re-evaporation of the liquid water occurs. Moreover, there is more liquid water condensing on the N95 FFR inner surface because it is subjected to direct exhaled airflow. Experimental results show that the measured relative humidity inside the FFR is approximately 1.5~2.6 times higher than that under the initial ambient condition of a temperature of 296.25 K and ambient relative humidity of 34.4%. These results indicate that the wet and warm micro-climate may contribute to bacterial growth. Experimental results of the bacterial distribution indicate that more bacteria exist on the inner surface of the FFR close to the human chin where a larger relative humidity will take place. The wetter and warmer region can be used to qualitatively estimate the bacterial distribution. In future work, we will study the microbes of patients with respiratory diseases.

## Data Availability

The datasets generated during and/or analysed during the current study are available from the corresponding author on reasonable request.
